# Autophagy stimulation delayed biological aging and decreased cardiac differentiation in rabbit mesenchymal stem cells

**DOI:** 10.34172/jcvtr.2021.43

**Published:** 2021-08-25

**Authors:** Mehdi Hassanpour, Omid Cheraghi, Reza Rahbarghazi, Mohammad Nouri

**Affiliations:** ^1^Student Research Committee, Tabriz University of Medical Sciences, Tabriz, Iran; ^2^Department of Clinical Biochemistry and Laboratory Medicine, Tabriz University of Medical Sciences, Tabriz, Iran; ^3^Department of Biochemistry, Faculty of Biological Science, Tarbiat Modares University, Tehran, Iran; ^4^Department of Applied Cell Science, Faculty of Advanced Medical Sciences, Tabriz University of Medical Sciences, Tabriz, Iran

**Keywords:** Bone Marrow Mesenchymal Stem Cells, Autophagy, Differentiation, Cardiomyocyte, Aging

## Abstract

***Introduction:*** Cardiovascular disease (CVD) is a type of disease that affects the function of cardiac-vascular tissues. This study aimed to consider the possible effects of autophagy, as an intrinsic catabolic pathway of cells, on the differentiation and aging process of mesenchymal stem cells (MSCs).

***Methods:*** In this study, bone marrow-derived MSCs were obtained from rabbit bone marrow aspirates. The stemness feature was confirmed by using flow cytometry analysis Cells at passage three were treated with 50 μM Metformin and 15μM hydroxychloroquine (HCQ) for 72 hours. The intracellular accumulation of autophagolysosomes was imaged using LysoTracker staining. Protein levels of autophagy (LC3II/I ratio), aging (Klotho, PARP-1, and Sirt-1) effectors, and cardiomyocyte-like phenotype (α-actinin) were studied by western blotting.

***Results:*** Based on our findings, flow cytometry analysis showed that the obtained cells expressed CD44 and CD133 strongly, and CD31 and CD34 dimly, showing a typical characteristic of MSCs. Our data confirmed an increased LC3II/I ratio in the metformin-received group compared to the untreated and HCQ-treated cells (*P* < 0.05). Besides, we showed that the incubation of rabbit MSCs with HCQ increased cellular aging by induction of PARP-1 while Metformin increased rejuvenating factor Sirt-1 comparing with the normal group (*P* < 0.05). Western blotting data showed that the autophagy stimulation response in rabbit MSCs postponed the biological aging and decreased the differentiation potential to the cardiac cells by diminishing α-actinin comparing with control cells (*P* < 0.05).

***Conclusion:*** In summary, for the informants in this study, it could be noted that autophagy inhibition/stimulation could alter rabbit MSCs aging and differentiation capacity.

## Introduction

Cardiovascular disease (CVD) is a type of disease that affects the function of cardiac and vascular tissues. Among the CVDs, acute myocardial infarction (AMI) is the principal reason of the fatality rate globally with socio-economical complications. Therefore, many efforts have been placed to develop new therapeutic strategies.^1,2^ Despite tremendous progress In AMI management, many people with CVD still develop chronic heart failure (CHF).^3^ Because of the complexities of existing approaches such as their after effects, deficiencies, and problems, there are many substitute approaches accessible such as drug administration, heart transplantation, and intra/extra cardiac devices to recover the quality of patient’s lifestyle. In most cases, these approaches are not efficient enough to restore the normal function of infarcted hearts. In recent decades, regenerative medicine as a substitute strategy paves a way to accelerate the restoration and recovery of injured tissues. This modality is applicable to AMI candidates.^4^ Based on data from in-vivo studies and clinical trial investigations, the application of multiple transplant cells in AMI candidates could restore the function of damaged cardiomyocytes and improve patient survival rate.^5^ In this regard, different cell sources are applicable to accelerate healing procedures in the cardiac tissue. For example, ESCs, CPCs, MSCs, EPCs, and iPSCs have been applied in different experimental studies.^6^ Stem cells are a specific population with a unique stemness feature, clonogenic capacity, prominent proliferation, and angiogenic behavior. Molecular investigations showed heterogenic expression of OCT4, KLF4) NANOG, SOX2, CD146, alkaline phosphatase, etc.^7^ The normal activity of stem cells and their regenerative potential are closely associated with diverse mechanisms. For example, it has been well-documented that the regenerative capability of different stem progenitors decreases by aging.^8,9^ Many factors and signaling pathways could decrease/increase the aging process in stem cells.^9,10^ The autophagy, along with ubiquitin-proteasome system, are believed to be the two main pathways for degradation of intracellularly disrupted substances.^11^ Autophagy is a complex intracellular pathway that carries and reduces damaged proteins / entire dysfunctional organs by fusion in lysosomes and the effects of hydrolytic enzymes.^12^ The close interplay between autophagy and aging procedure has been shown previously.^13^ The promotion of autophagic response could dictate different regenerative potential in pericytes and other stem cells.^14^ In this respect, Zhang et al reported that autophagy induction in CPC increases differentiation into functional cardiomyocytes, and shows the main character of the autophagy flux in the ancestral differentiation.^15^ Mounting documents acclaimed that autophagy have a critical function in aging process and aging-associated diseases.^16-18^ Induced autophagy may postpone senescence, resulting in prolonged life-span.^19^ The dysfunctional autophagic cundition caused the gathering of impaired materials inside the cell, causing advent of aging-related abnormalities.^20^ So, it is remarkable to extend strategies to prolong the life-span by the autophagy modulation strategies. Despite multiple stem cell types’ effectiveness in numerous essential biological and physiological processes like angiogenesis, migration, differentiation, and maturation of functional heart cells. There have been few reports of the effects of autophagy modulation on stem cell differentiation and aging process. In the present study, our aim was to assess the possible effects of autophagy modifications on the differentiation and aging potential of MSCs.

## Materials and Methods

### Purification of bone marrow mononuclear cells (MNCs)

**Figure 1:**
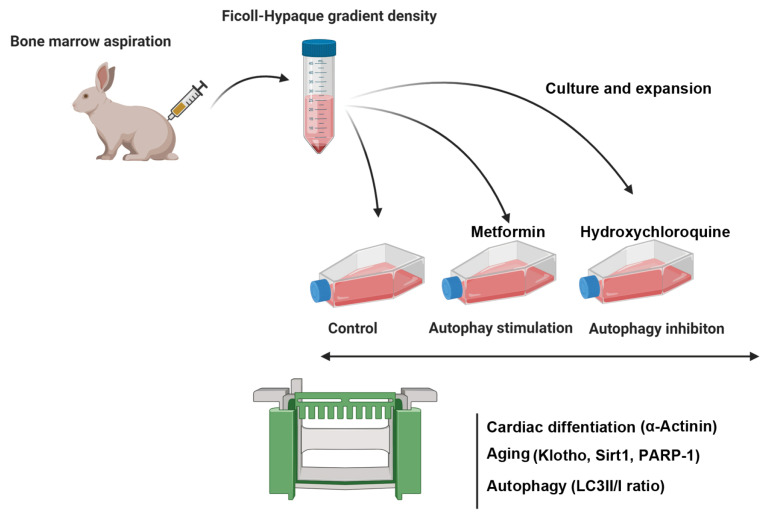
Graphical abstract of the study

This study has been permitted by the Ethics Committee, Tabriz University of Medical Sciences (IR.TBZMED.VCR.REC.1397.132). At present study, two matured white rabbits (New Zealand race) were preserved in separate cages. After animal anaesthetization (35 mg/kg bw ketamine/5 mg/kg bw xylazine), the bone marrow blood content of femur was aspirated by Jamshidi needle containing 1000 IU/ml sodium heparin. Following dilution of obtained bloods with (volume ratio 1:1), MNCs were isolated using Ficoll-Hypaque protocol according to our previous study.^21^ To this end, the prepared bloods were gradually overlaid on the Ficoll-Hypaque solution (Sigma-Aldrich) and centrifuged at 450*g* for 25 min. Afterwards, layer containing MNCs was gathered at between the plasma and the Ficoll solution. After washing with PBS, MNCs suspended in DMEM/LG medium (Gibco), supplemented with %5 FBS (Gibco). The exhausted culture mediums were changed every 4 days (Figure 1).

### Immunophenotyping of rabbit MSCs by flow cytometry analysis

The surface markers of adherent cells were analyzed by flow cytometry evaluation, using antibodies directed to MSC related antigens such as PE-conjugated anti-rabbit CD44 (ebioscience), FITC-conjugated anti-rabbit CD31 (ebioscience), CD34 (ebioscience), CD133 (ebioscience). In brief, cell suspension harvested after treatment by 0.025% Trypsin–EDTA solution (Gibco), blocked with 1% BSA for 30 min, and exposed to antibodies as said by manufacturer’s instruction. Besides, appropriate isotype normal antibodies were applied to eliminate background staining. Next, the cell suspension was washed by PBS, treated with 4% paraformaldehyde as a fixative agent, and examined by the flow cytometer. Lastly, the obtained results were evaluated via FlowJo software version 8.6.1.

### LysoTracker assessment

To evaluate the inhibitory effects of hydroxychloroquine (HCQ) on autophagy status, LysoTracker staining was performed according to our previously published work.^22^ For this purpose, rabbit MSCs were cultured at Chambered Slide (SPL) (10000 cells in each well) and kept at incubator condition for 24 h. The next day, MSCs were exposed to HCQ (15 and 20 μM) for 3 days. When the incubation period was completed, MSCs were incubated with 50 nM LysoTracker solution (Sigma-Aldrich) for 30 min. For nuclear staining, MSCs were treated with a 1 μg/mL diamidino-phenylindole (DAPI) (Sigma-Aldrich) for 30 min. The trapping intracellular vacuoles of MSCs were imaged by immunofluorescence microscopy.

### Modulation of autophagy flux

In current work, we examined the possible effects of autophagy modifications on differentiation capacity and aging of MSCs. MNCs were cultivated in DMEM supplemented media with 1% FBS solution and incubated for 7 days. After 4 days, autophagic flux of cultured cells was induced/inhibited by 50 µM Metformin (Met) (Cat no: Osveh Pharmaceutical Inc., Iran) and 15 µM HCQ (Sigma-Aldrich). After 7 days, cells were harvested and western blotting was performed.

### Morphological changes after autophagy modulation

Three days after incubation with Met and HCQ, we monitored the morphological changes by using inverted microscopy.

### Western blot analysis

Western blotting was performed to analyze the autophagy-, cardiomyocyte-, and aging-related proteins levels in MSCs after autophagy modulation. After cell collection on 7^th^ day, cells were lysed in lysis buffer solution supplemented with cocktail inhibitors, sonicated at a 50 Hz, and centrifuged at 13000 g for 30 min. Then, total protein concentration was quantified by the spectrophotometric system. Then, 100 µg of proteins from each group were overloaded into the wells of the 12% SDS-PAGE gel and relocated to the PVDF membrane, along with molecular weight markers. The PVDF membranes were exposed in antibody solution at 4°C overnight (Beclin-1: Santa Cruz Biotechnology, Inc; LC3: Abcam; P62: Santa Cruz Biotechnology, Inc; α-actinin-2: Santa Cruz Biotechnology, Inc.; PARP-1: Santa Cruz Biotechnology, Inc.; Silent information regulator 1 (SIRT1): Santa Cruz Biotechnology, Inc.; Klotho (E-21): Santa Cruz Biotechnology, Inc.). Next, the membranes were exposed to the secondary HRP-conjugated anti-IgG antibody (Santa Cruz Biotechnology, Inc) for 60 min at RT. The immunereactive spots were identified using the ECL solution (BioRad). The immunoreactive spots were considered with ImageJ software version 1.44p. For data normalization, β-actin antibody (Santa Cruz Biotechnology, Inc) was applied as a housekeeping protein. The experimentation was done 3 times.

### Statistical analysis

The statistical analysis was completed by Graph Pad software (Ver Prism 9). All quantitative statistics were provided as mean ± standard deviation (SD) and Student t-test applied for analysis. Statistically significant levels were reflected at *P* <  0.05.

## Results

### Flow cytometry analysis of isolated rabbit MSCs

**Figure 2:**
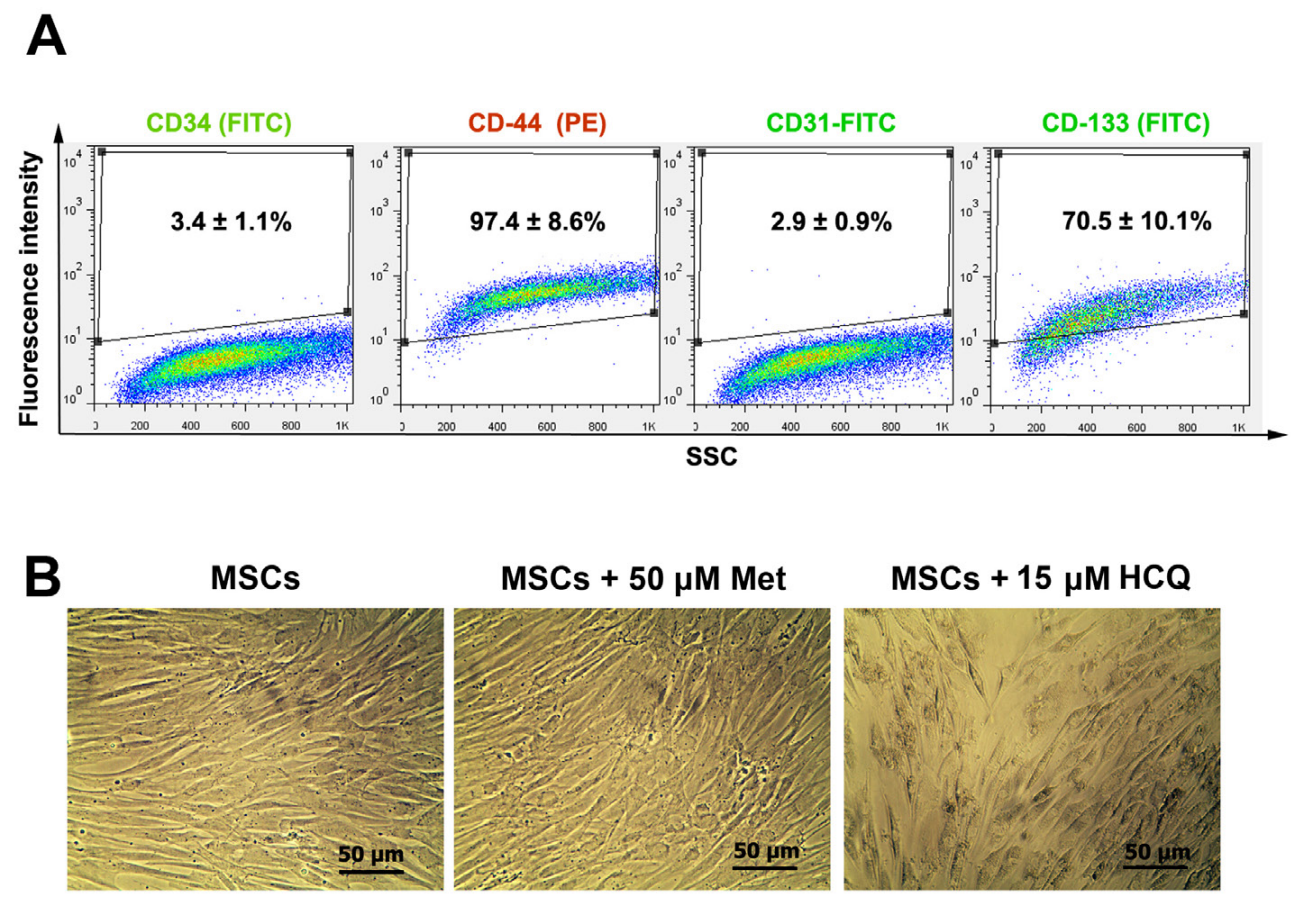
Flowcytometric immunophenotyping of MSCs showed positive staining for CD44 and CD133 and relatively positive staining for CD31 and CD34 (A). Morphology of rabbit MSCs after treatment with Met and HCQ showed that the inhibition of autophagy with 15 µM HCQ change the morphology of MSCs (B)

Flow cytometry analysis was done to immunophenotype the expanded cells (Figure 2A). Data showed that the isolated cells were positive for CD44 (97.4 ± 8.6%), CD133 (70.5 ± 10.1%) and relatively express CD31 (2.9 ± 0.9%) and CD34 (3.4 ± 1.1%). These data demonstrated that the majority of cells possess characteristics similar to MSCs.

### Morphological changes

Monitoring morphology of rabbit MSCs after treatment with Met and HCQ showed that autophagy modulation could change the cell shape (Figure 2B). In normal condition, undifferentiated MSC exhibited spindle shaped morphology which is typical to mesenchymal lineage. Treatment of rabbit MSCs with Met did not alter cell morphology while the exposure of these cells with 15 µM HCQ disrupted the cell-to-cell connection and contribute to formation of discrete or round cells. These data revealed that inhibition of autophagy in stem cells could alter cell morphology.

### HCQ treatment contributed to the accumulation of intracellular vacuoles inside rabbit MSCs

**Figure 3:**
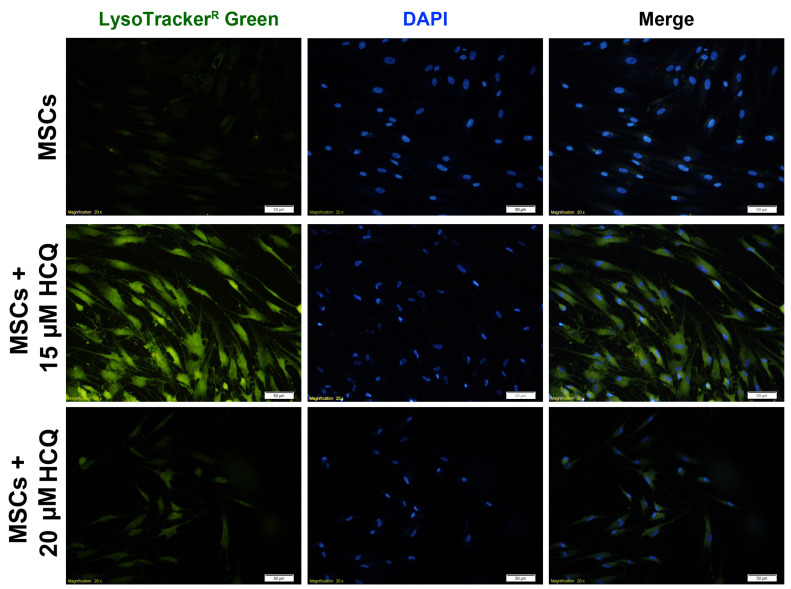
The result of LysoTracker Green staining illustrated that 15-μM HCQ is the optimum dose for autophagy inhibition in MSCs with a minimal toxic effect

To confirm autophagy suppression in rabbit MSCs, the LysoTracker staining was performed (Figure 3). According to our data, the number and intensity of LysoTracker^+^ MSCs with intracellular lysosomes increased in 15 μM HCQ group comparing to the non-treated control group. In group 20 μM HCQ, we found LysoTracker+ MSCs was less than the 15µM HCQ group. Our results demonstrated that a 72 h exposure of rabbit MSCs with HCQ, especially 15 μM block the flux of vacuoles and contributed to accumulation of lysosomes inside the MSCs. According to this data, we designated 15 μM HCQ for following analyses.

### Autophagy stimulation decreased rabbit MSCs differentiation capacity and reduced aging

**Figure 4:**
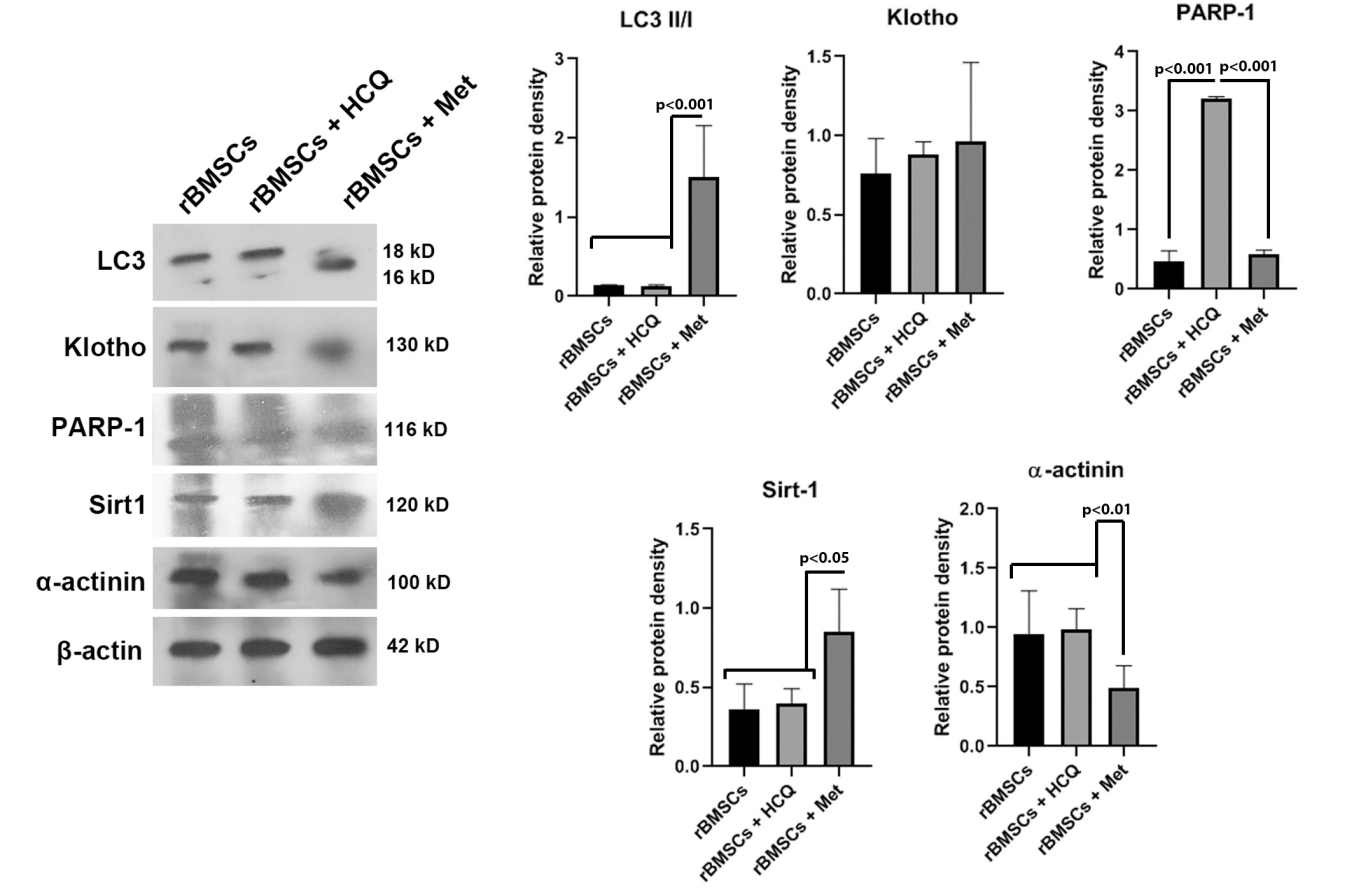
The results of western blotting autophagy-related factor (LCII/I) aging-related factor (Klotho, PARP-1 and Sirt-1) and differentiation to cardiogenic lineage (α-actinin) demonstrate the potency of Met in the induction of autophagy via the increase of LC3-II/I ratio. These data showed that the promotion of autophagy response in rabbit MSCs postponed the biological aging and decreased the differentiation potential to the cardiac cells

Primary MSCs were cultivated and exposed to 50 µM Met as autophagy inducer and 15 µM HCQ as autophagy blocker for 3 days *in vitro*. The protein analysis of different factors related to anti-aging (PARP-1, SIRT1, and Klotho), autophagy (LC3-II/I), and differentiation to cardiogenic lineage (α-actinin) was done via using western blotting. Our data confirmed the potency of Met in the induction of autophagy via the increase of LC3-II/I ratio while this value remained unchanged in the MSCs exposed to the HCQ (Figure 4). Data showed that the stimulation of autophagy coincided with the increase of Klotho and Sirt-1 while the level of PARP-1 aging and maturation factor was diminished. By increasing the activity of Klotho, and Sirt-1, the potency of rabbit bone marrow MSCs to trans-differentiate into cardiac lineage was also increased. These data illustrated that the enhancement of autophagy response in rabbit MSCs postponed the biological aging and decreased the differentiation potential to the cardiac cells.

## Discussion

Up to date, many efforts have been done to find an alternative approach for the amelioration of AMI in addition to conventional therapeutic approaches.^23^ It seems that stem cells are novel and reliable cell source to be used in different pathological conditions.^24^ The main issue with the use of stem progenitors in the target tissues is the cell number and dosage required for therapies.^25^ According to previously published data, a large number of transplant cells die shortly after transplantation into the target sites because of inappropriate handling procedure and lack of extrinsic prosurvival signals.^26^ These condition contribute to limited capability of stem cells for successful engraftment.^26,27^ It has been shown that autophagic degradation flux, is an early cell reaction in response to different external and internal insults which may protect cells from subsequent injuries.^28^ The inhibition of autophagic response could lead to the accumulation of intracellular exhausted cargoes and subsequent injuries.^28^ In addition, in a growing scientific literature, the potential effects of autophagy on the differentiation capability and phenotype of different stem progenitors has been well established.^29,30^ Here, we aimed to show the inhibition and stimulation of autophagic response in rabbit MSCs differentiation and aging. We show that the incubation of rabbit MSCs with HCQ did increase the number of autophagolysosomes after 3 days, which indicates inhibition of autophagic flow.^31^ In contrast, 50 µM Met increased the LC3II/I ratio, indicating enhanced autophagic response.^32^ There are some little investigations that clarify link of autophagy and aging. In addition, we also monitored the levels of factors participating in cellular aging. Based on our data, autophagy stimulation by 50 µM Met increased protein levels of Klotho and Sirt-1 and decreased PARP-a levels compare to group incubated with by 15 µM HCQ, proposing that autophagic condition have a critical role in the senecence of rabbit MSCs. In line with our study, previously, several study declared that autophagy induction by rapamycin prolong lifespan in many organisms.^19,33,34^ We also monitored the possible effect of autophagy on cardiac cell differentiation of rabbit MSCs by monitoring α-actinin after being treated with HCQ and Met. Our data showed a significant reduction of α-actinin in Met-treated mesenchymal stem cells compared with untreated and HCQ groups. These results suggest that induction of autophagy could reduce differentiation potential of rabbit MSCs toward cardiomyocyte-like cells. Contradiction with our data, the incubation of T regulatory lymphocytes with rapamycin, an autophagy stimulator, did not alter maturation and differentiation capacity.^35^ Previous data confirmed controversial effects of autophagy on differentiation capacity of stem cells.^35^ Recently, we have demonstrated that autophagy flux significantly increase differentiation of CD146^+^ cells into cardiac cells, including mature pericytes, functional endothelial cells and cardiomyocytes.^14^ Moreover, Gupta et al declared that autophagy inhibition drastically reduces α-smooth muscle actin (α-SMA) protein in cardiac fibroblasts comparing to non-treated control. Further examinations are obligatory to clarify the dual effects of autophagic status on stem cell differentiation toward different lineage. Numerous discrepancies may be due to the fact that the cell type, incubation time are different in the most of these studies. We exhibited that the induction of autophagic status in stem progenitors could decrease cellular aging by the increase of Sirt-1. It has been shown that this factor participates in the rejuvenation of cells.^36^

Based on the results of present work and recently published documents, it could be concluded that autophagy modulation in stem cells like MSCs could exemplify a novel therapeutic methodology that could improve efficiency of MSCs in the management of various diseases.^37^ Remarkably, conflicting results on the consequences of autophagic flux modulation in MSCs suggest that caution should be exercised and further in vitro and in vivo research prior to the use of autophagy-modulated MSCs in clinical setting examinations.

## Conclusion

Based on our observations, in another general note, we can state that the stimulation of autophagy by 50 μmol Met, by modulating Klotho, PARP-1 and Sirt-1, has the potential to differentiate rabbit mesenchymal stem cells into quasi-lineage Reduces the heart, which leads to suppression of the aging process of cells. According to the note, cell treatment with HCQ autophagy blocker does not alter the differentiation capacity while increasing cellular aging. Whether autophagy modulation pre-determined cell differentiation into the specific lineage is subject of debate. There are numerous data showing the contradictory results after the autophagy modulation in the differentiation capacity of the productive cell. This controversies would be related to species, incubation time, dose of modulators, and the target molecules monitored. It is also suggested that autophagy can control the cell differentiation capacity in early stage rather than the late stages.^38^ It is not yet known how cells can continuously respond to autophagic modulation at different stages of growth.

## Acknowledgements

All authors express appreciation to the Student Research Committee, Tabriz University of Medical Sciences, Tabriz, Iran.

## Competing interests

The authors declare no conflict of interest in this study.

## Ethical approval

The study protocol was permitted by the ethical committee of TUOMS (IR.TBZMED.VCR.REC.1397.132).

## Funding

Student Research Committee, Tabriz University of Medical Sciences, Tabriz, Iran (Grant No: 60247).
